# The maternal microbiome: another bridge linking mothers and infants

**DOI:** 10.1016/j.ebiom.2021.103602

**Published:** 2021-09-24

**Authors:** EBioMedicine

A total of 10–100 trillion microbes live symbiotically within each human host and are thought to affect our physical and mental health. The health effects are thought to begin as early as the gestational period. Research shows that maternal gut microbes may have both direct and indirect effects during pregnancy. For example, a dysregulated gut microbiome is thought to promote intestinal inflammation, which in turn could lead to a shortening of the gestational period and a reduction in birthweight. The gut microbiome can also influence nutrient absorption during pregnancy and cause more global effects on gestation and fetal growth.

Some adverse pregnancy outcomes, such as preterm birth and low birthweight, are more prevalent in low-income and middle-income countries (LMIC). Despite the important role of maternal gut microbes on these outcomes, most studies to date have been done in high-income populations. This misalignment urges more studies on maternal gut microbiome in LMICs. Recently in June issue of *EBioMedicine*, Ethan Gough and colleagues reported the relationship between the maternal faecal microbiome and gestational age, birthweight, and neonatal growth in rural Zimbabwe. They found that *Blastoscystis sp, Brachyspira sp*, and *Treponeme* carriage were higher in this Zimbabwe cohort than populations in high-income countries. Resistant starch-degraders were the predominant finding and were important predictors of birth outcomes. Zimbabwean mothers included in this study consumed diets that were high in maize. It's thought that the resistant starch-degraders could help to release energy from polysaccharides in the maize that cannot be digested by host enzymes and therefore provide an important nutrient-harvesting function for these mothers. The study also investigated the metabolic pathways and enzymes present in the maternal gut microbiome and found that pathways related to environmental sensing, vitamin B metabolism, and signalling were associated with **i**ncreased infant birthweight and better neonatal growth, while those related to functions involved in biofilm formation in response to nutrient starvation predicted reduced birthweight and worse growth.

In addition to influencing neonatal growth, evidence indicates that the maternal gut microbiome also affects infant psychological development. As early as 2016 in *Cell*, Shelly Buffington and colleagues showed that a high-fat diet induced a shift in maternal gut microbiota in a mouse model, especially the lowered abundance of *Lactobacillus reuteri*, which reduced oxytocin levels in the hypothalamus of the offspring and negatively affected their social behaviour. Supporting this animal study, in the June issue of *EBioMedicine*, Samantha Dawson and colleagues found that taxa from butyrate-producing families *Lachnospiraceae* and *Ruminococcaceae* were more abundant in mothers of children with normative behaviour. A healthy prenatal diet, including a high intake of fish, nuts, eggs, green vegetables, whole grains, and a low intake of white bread, sugar, full-cream milk, and hamburgers, indirectly related to decreased internalising behaviour in children via higher alpha diversity of maternal faecal microbiota. Although further studies are needed to delineate the underlying mechanisms, this study provides early evidence that maternal prenatal gut microbiota might affect the psychological development of children and could be helpful for obstetricians and nutritionists to inform the diets of pregnant women.

The maternal microbiome not only affects various neonatal outcomes and their early development externally, but can also be directly seeded into infant guts to influence their health internally. It was long believed that infant gut microbes are seeded during labour, particularly from the maternal vaginal microbiome, as infants born by caesarean section showed measurably different features of gut microbiota in early childhood, compared with infants who are born vaginally. In particular, a low abundance of *Bacteroides* has been observed in infants born by caesarean section. However, this notion is challenged by recent evidence. In the December 2020 issue of *Cell Reports Medicine*, Caroline Mitchell and colleagues compared the microbial profiles of infants who were vaginally born or infants born by planned caesarean section with infants born by emergent caesarean section who were exposed to the maternal vaginal microbiome but eventually delivered by caesarean section. They found that even though 33 (94.3%) out of 35 children born by caesarean section had detectable levels of *Bacteroides* in the first few days of their lives, both planned and emergent caesarean section groups lacked *Bacteroides* at 2 weeks. After comparing the microbial strain profiles between infants and maternal vaginal or rectal samples, the authors confirmed the mother-to-child bacterial transmission occurred during vaginal delivery, but the maternal source was rectal rather than vaginal. Furthermore, this vaginal origin theory was also questioned by a pilot randomised placebo-controlled clinical trial, reported in the July issue of *EBioMedicine*, which aimed to restore gut microbiome development in infants born by caesarean section by oral administration of maternal vaginal microbes. The authors observed no differences in gut microbiome composition or functional potential between infants born by caesarean section receiving either maternal vaginal microbes or sterile water (placebo). Compared with infants born by vaginal delivery, the gut microbiomes of both caesarean section groups showed the characteristic feature of low *Bacteroides* abundance with several biosynthesis pathways being underrepresented.

Human microbiota is a complicated ecosystem, influenced by a variety of personal and environmental factors. What is certain is that the maternal microbiome influences the pregnancy, foetal development, and infant health. However, the detailed mechanisms are still largely unknown. Considering the diversity of the microbes and individuals, future studies with rigorous designs and a large cohort size are warranted. To improve equity, populations with different ethnic and socioeconomic backgrounds should be considered as well.

